# Chemical Composition, Antioxidant, and Cytotoxic Activity of Essential Oils in the Above-Ground Parts of *Sonchus oleraceus* L.

**DOI:** 10.3390/plants13121712

**Published:** 2024-06-20

**Authors:** Abd El-Nasser G. El Gendy, Nadia A. Mohamed, Tushar C. Sarker, Emad M. Hassan, Ahmed H. Garaa, Abdelsamed I. Elshamy, Ahmed M. Abd-ElGawad

**Affiliations:** 1 Medicinal and Aromatic Plants Research Department, National Research Centre, Giza 12622, Egypt; ag-gundy@nrc.sci.eg (A.E.-N.G.E.G.);; 2 Medical Biochemistry Department, National Research Centre, Cairo 12622, Egypt; 3 Texs A&M AgriLife Research Center, Overton, TX 75684, USA; tushar.sarker@ag.tamu.edu; 4 Department of Natural Compounds Chemistry, National Research Centre, Giza 12622, Egypt; 5 Plant Production Department, College of Food & Agriculture Sciences, King Saud University, P.O. Box 2460, Riyadh 11451, Saudi Arabia

**Keywords:** *Sonchus oleraceus*, volatile components, chemical profile, trans-caryophyllene, antioxidant, anticancer

## Abstract

*Sonchus oleraceus* L. is a leafy vegetable that is usually consumed in the area of the Mediterranean and is a frequently used traditional herb to treat a variety of ailments. Previous studies deduced the potent antioxidant and cytotoxic functions of the different extracts and isolated compounds from *S. oleraceus*. The current study represents the first instance of chemical profiling and bioactivities of the extracted essential oil (EO) of *S. oleraceus*. The present investigation set out to identify the chemical components of this EO by means of Gas Chromatography with Flame Ionization Detector (GC-FID) and Gas Chromatography-Mass Spectrometry (G004-MS) techniques; assess the oil’s antioxidant potencies through 1,1-diphenyl-2-picrylhydrazyl (DPPH) and 2,2′-azinobis-(3-ethylbenzothiazoline-6-sulfonate (ABTS) assays; and evaluate the oil’s cytotoxic impact against HepG2 cancer cell lines. The GC-MS chemical profiling revealed the identification of 23 components representing 97.43% of the total oil mass within abundant cyclic ketones (20.15%), nonterpenoidial hydrocarbons (28.77%), and sesquiterpenes (42.19%). The main components were *n*-nonadecane (28.77%), *trans*-caryophyllene (23.73%), *trans*-methyl dihydrojasmonate (19.55%), and *cis*-cadina-1,4-diene (9.44%). In a dose-dependent manner, this EO demonstrated antioxidant capacities on DPPH and ABTS, with IC_50_ values of 609.35 and 804.16 µg/mL, respectively, compared to ascorbic acid. Using doxorubicin as a reference therapy, the MTT assay findings revealed that this oil had remarkable inhibitory effects on the proliferation of HepG2 cancer cell lines, with an IC_50_ of 136.02 µg/mL. More studies were recommended for further investigation of new biological roles for this oil and its main components, along with the construction of action mechanisms based on chemical components.

## 1. Introduction

Using plants therapeutically to treat a variety of illnesses is an old practice that has recently regained popularity. Medicinal plants provide more than half of the drugs used in the therapeutic treatment of cancer [1].

Essential oils (EOs) possess a volatile, fragrant, and highly complex mixture of mainly terpenoid components. Monoterpenes, sesquiterpenes, and aromatic compounds constitute the vast majority of the mixture. Steam distillation is the primary method used to deliver the EOs [2]. EOs show a range of activities, including anticancer effects [3]. Numerous EOs have been found to have antioxidant qualities, and their application as a natural antioxidant is growing in popularity due to current worries about possible health risks connected to some synthetic antioxidants. For food goods, adding EOs directly through mixing, active storage, or food-safe coatings could be a good substitute to stop autoxidation and increase shelf life [4,5].

The Asteraceae family is one of the largest flowering plant groups in the world, comprising roughly 1600 genera and 2500 species worldwide. Its most well-known taxa are lettuce, artichokes, chicory, daisies, and dandelion. People have used Asteraceae plants for food and medicine for millennia [6,7]. Due to their unique phytochemical components, which include terpenes, polyphenolic compounds, lignans, saponins, sterols, and polysaccharides in addition to EOs, some of these taxa exhibit a variety of pharmacological characteristics [7].

Sowthistle, or *S. oleraceus* L., is a leafy vegetable that is commonly eaten in Mediterranean countries [8]. Its leaves and soft, fresh stems are the only edible portions of *Sonchus oleraceus* (Asteraceae) that can be added to stews or salads [8]. The aerial parts of this plant have been utilised by herbalists in Cameroon for the treatment of arthritis, infections, inflammation, headaches, diabetes, stomach pain, liver infection, and general nuisances [9,10]. *S. oleraceus* has a wide range of biological characteristics that have been investigated previously and documented, especially anti-inflammatory, anti-diabetic, and antioxidant activities [9,11,12]. There are no published studies on the chemical composition and/or biological characteristics of EO derived from *S. oleraceus*.

Therefore, the present study deals with (i) the identification of the chemical composition of the EO, derived by the hydro-distillation of the above-ground parts of *S. oleraceus* via Gas Chromatography with Flame Ionization Detector (GC-FID) and Gas Chromatography-Mass Spectrometry (GC-MS) techniques; (ii) the assessment of antioxidant activity against the DPPH and ABTS free radicals; and (iii) the evaluation of the cytotoxic activity of this oil against liver (HepG2) cancer cell lines.

## 2. Material and Methods

### 2.1. Plant Materials

**Figure 1:**
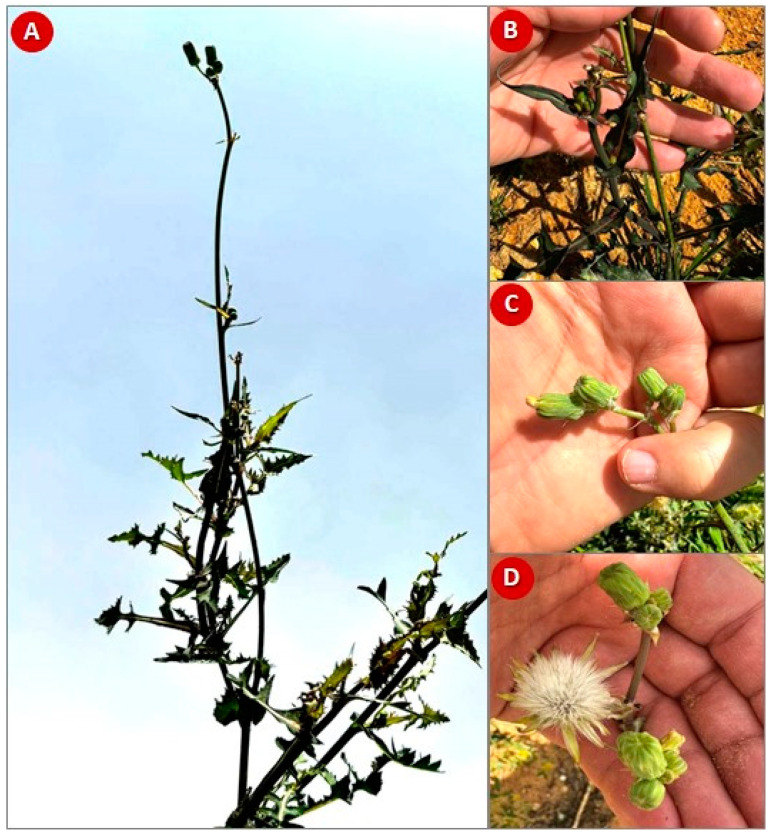
The herb *Sonchus oleraceus* L. (**A**) Plant shoot system, (**B**) close view of leaves, (**C**) close view of closed inflorescence (head), and (**D**) open inflorescence.

Above-ground parts of *S. oleraceus* (Figure 1) were collected in May 2021 throughout the plant’s flowering period from the Nile Delta region of Menoufia (30.3502780332799, 30.869978698312085), Egypt. Dr. Ahmed Abd ElGawad, a taxonomy professor from Mansoura University, verified the identity of the plant. A voucher specimen (SO-xq-774z/21-02712) was stored in the Herbarium of the Faculty of Science, Mansoura University.

### 2.2. Isolation of the EO

*Sonchus oleraceus*’s (200 g) fresh above-ground portions were chopped and then hydro-distilled in a 2 L round flask employing Clevenger apparatus. Following three hours of hydro-distillation, the plant’s extracted EO was poured out, sorted, and quickly dried with 0.5 g of anhydrous sodium sulphate. Three separate plant samples, each weighing 200 g, underwent the same procedures. The three extracted EO samples were stored in three different vials of glass in the refrigerator until the starting point of the chemical and biological analyses. To find the EO yields (*v*/*w*%), the subsequent formula was utilised:

$$Yield\;of\;EO\%=\frac{volume\;of\;derived\;EO}{extracted\;plant\;weight}\times 100$$

### 2.3. Gas Chromatography with Flame Ionization Detector (GC-FID) and Gas Chromatography-Mass Spectrometry (GC-MS) Analysis

The isolated EO samples were analysed according to the same protocols described before by Abd-ElGawad et al., 2023 [13] and El Gendy et al., 2024 [14]. In brief, The GC-FID analysis was performed using an HP-5890 series II apparatus (Hewlett-Packard, Palo Alto, CA, USA) set with two silica capillary columns (30 m 0.25 mm; film thickness: 0.25 m), HP-Wax, and DB-5 (Agilent, Santa Clara, CA, USA). The temperature of the oven was planned to rise from 60 °C to 220 °C at a rate of 5 °C per minute (with the carrier gas N_2_ at 2 mL/min; spitless injecting), with the injector and detector set at 250 °C.

The apparatus used for the Gas Chromatography-Mass Spectrometry (GC-MS) analysis was an ISQTM Single Quadrupole Mass Spectrometer acting as the detector and a TRACE GC Ultra Gas Chromatograph from ThermoFisher Scientific, located in Waltham, MA, USA. A TR-5 MS column with a length of 30 m, an inner diameter of 0.32 mm, and a film thickness of 0.25 μm was fitted with the GC-MS system. Helium was employed as the carrier gas, flowing at a rate of 1.0 mL min^−1^, with a split ratio of 1:10 until 240 °C was attained, following which the temperature increased at a rate of 4.0 °C per minute for a duration of one minute from 60 °C. At 210 °C, the injector and detector were maintained. The injections employed 1 μL of diluted EO samples in the mixes (1:10 hexane, *v*/*v*). Electron ionisation (EI) at 70 eV and the spectral range of *m*/*z* 40–450 were used to acquire mass spectra.

The identity and verification of the chemical components were performed using the NIST database, Wiley spectrum collection, AMDIS software (Automated Mass Spectrum Deconvolution and Identification; version 2.7) [15], and retention indices of *n*-alkanes (C_8_–C_22_). In addition, the constituents were identified by comparing the components’ retention times with those of genuine samples using the components’ linear retention indices in relation to the *n*-hydrocarbon sequence and computer matching against commercial and homemade library mass spectra composed of known oil components, authentic mass spectra, and MS literature data. For GC-MS analysis, the following compounds were used as genuine examples: camphene, (α- and β-) pinene, D-limonene, linalool, α-terpineol, geraniol, citral, α-elemene, α-copaene, β-germacrene D, caryophyllene, cubedol, spathulenol, and α-eudesmol.

### 2.4. Free Radical Scavenging Assessments

The 1,1-diphenyl-2-picrylhydrazyl (DPPH) and 2,2′-azinobis-(3-ethylbenzothiazoline-6-sulfonate (ABTS) assessments were utilised for evaluating the antioxidant capacities of the EO derived from above-ground sections of *S. oleraceus* for the first time. The EO was diluted with methanol to obtain the various concentrations. Thus, for this purpose, five concentrations—62.5, 125, 250, 500, and 1000 µg mL^−1^—were developed. Similarly, five vitamin C concentrations (25.0, 50.0, 75.0, 100.0, and 125.0 µg mL^−1^) were prepared as a standard antioxidant drug by dissolving and diluting it with methanol.

#### 2.4.1. DPPH Assay

The ability of the isolated *S. oleraceus* EO to scavenge DPPH radicals was investigated according to the reported method by Miguel et al., 2010 [16]. A variety of EO concentrations of 62.5, 125, 250, 500, and 1000 µg mL^−1^ were produced using ethanol as the solvent. After preparing equal parts of freshly synthesised DPPH (0.3 mM) and EO, they were vigorously mixed and left to stand at room temperature in the dark for 20 min. The absorbance at 517 nm was then found using a spectrophotometer (Analytik Jena, Jena, Germany).

#### 2.4.2. ABTS Assay

The antioxidant capacity of *S. oleraceus* EO was evaluated following the established method based on the reduction in ABTS [17]. The EO concentrations ranged from 65.5 to 1000 µg mL^−1^ and were produced in ethanol in accordance with the DPPH test protocol. In summary, the mixture was incubated for 6 min in the dark after 2 mL of freshly produced ABTS and 0.2 mL of each oil concentration were added. Utilising the Spectronic 21D spectrophotometer (Milton Roy, CA, USA), the colour absorbance at 734 nm was determined. Moreover, positive control with a dosage ranging from 12.5 to 225 µg mL^−1^ was made using vitamin C. For the DPPH and ABTS, the percentage that c referred to scavenging performance was established with the following formula:

$$Antioxidant\;\%=\left(\;1-\frac{A_{oil}}{A_{cont.}}\;\right)\times 100$$

where A_oil_: absorbance of the oil samples, and A_cont_.: absorbance of control.

### 2.5. Cytotoxic Activity of S. oleraceus EO

#### 2.5.1. Cell Culture

The cytotoxic activities of *S. oleraceus* EO were evaluated against the growth of the human cancer cells HepG2 (human hepatocellular carcinoma) (ATCC, Manassas, VA, USA). The cell type was cultivated at 37 °C in a 5% CO_2_ humidified atmosphere using MEM medium supplemented with 10% foetal bovine serum, 100 U/mL penicillin, and 100 g/mL streptomycin [18].

#### 2.5.2. MTT Assay

Using the MTT (3-[4,5,[4,5-dimethylthiazole-2-yl]-2,5-diphenyltetrazolium bromide) assay [19], the cytotoxic activity of the *S. oleraceus* EO against the human cancer cell, HepG2, was examined. EO concentrations of 0.1, 1.0, 10.0, and 100 µg/mL were created when 5 × 10^4^ cells/mL were seeded onto the 96-well microplate with 100 µL of culture media supplemented with 10% foetal bovine serum for the tests and then the mixture was incubated for 18–24 h at 37 °C with 5% CO_2_. Following the incubation period, the culture medium was disposed of, and 100 μL of the culture medium containing the measured concentrations of each EO concentration was incubated for 48 h at 37 °C with 5% CO_2_. Following incubation, the culture medium was disposed of, and 100 μL of the culture medium containing 0.5 mg/mL MTT was added to each well. The wells were then incubated for four hours at 37 °C with 5% CO_2_. Following the incubation period, the culture media was disposed of, and 100 μL was added to each well to lyse the cells. Using a microplate reader Model 680 (BioRad, Hercules, CA, USA), the absorbance of the converted dye was measured at 570 nm with background subtraction at 620 nm. The following formula was used to determine the percentage of cell viability and inhibition:

$$Cell\;viability=\frac{A\;treated}{A\;control}$$

where A treated: absorbance of the treated cells; and A control: the absorbance of control cell.

$$Inhibition\;\%=1-\frac{A\;treated}{A\;control}$$

IC_50_: an oil concentration that inhibited cell growth by 50%.

The studies were carried out in triplicate, and Prism Graphpad (version 5.0) software was utilised to compute the analytical parameter, which was 50% inhibition of cell growth (IC_50_).

## 3. Results and Discussion

### 3.1. S. oleraceus EO Chemical Profiling

**Figure 2:**
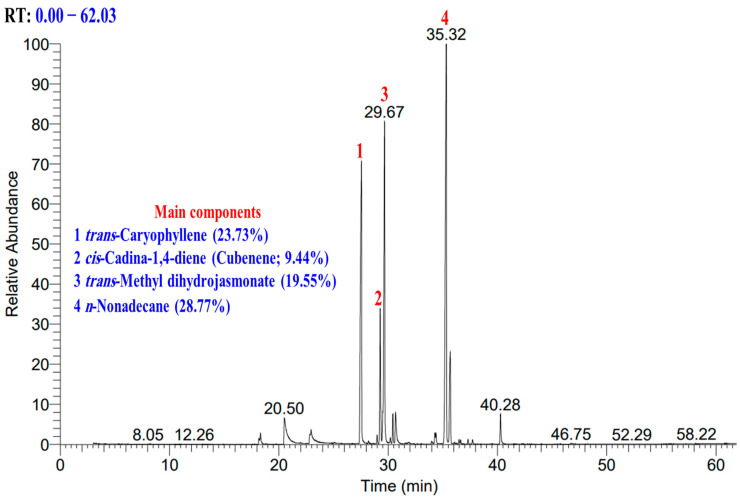
Total ion chromatogram (TIC) of EO derived from *S. oleraceus’s* above-ground parts.

The hydro-distillation of *S. oleraceus* plant’s above-ground parts afforded viscous oil with a pale-yellow colour and an overwhelming scent. The oil yield was 0.49% *v*/*w*. The plants belonging to the family Asteraceae were characterised by a high yield of EOs such as *Achillea collina* Becker ex Heimerl s.l. (0.73%, *v*/*w*) [20], *Artemisia sieberi* Besser (0.56–1.02, *v*/*w*) [21], *Achillea fragrantissima* (Forssk.) Sch.Bip. (0.84–0.96%, *v*/*w*) [22], and *Pulicaria undulata* L. (0.36–0.43%, *v*/*w*) [23]. The current study’s EO yielding was found to be higher than *S. arvensis* subsp. uliginosus’s oil yielding (0.002%) [24]. The quantitative and qualitative analysis of the extracted EO was carried out via GC-FID and GC-MS. Figure 2 presents the total ion chromatogram (TIC) of the analysed EO, along with the main components.

Based upon these analyses, the chemical volatile constituents were identified and are listed in Table 1 along with their retention times (Rt.) and relative concentrations (Rel. Conc.), in addition to reported and calculated values of Kovats indexes (KIs). Twenty-three compounds were identified, representing 97.43% of the overall oil mass. Ten classes of compounds were characterised, including benzenoids, cyclic ketones, fatty acid esters, hydrocarbons, oxygenated hydrocarbons, monoterpene hydrocarbons, oxygenated monoterpenes, sesquiterpene hydrocarbons, oxygenated sesquiterpenes, and carotenoid-derived compounds.

**Figure 3:**
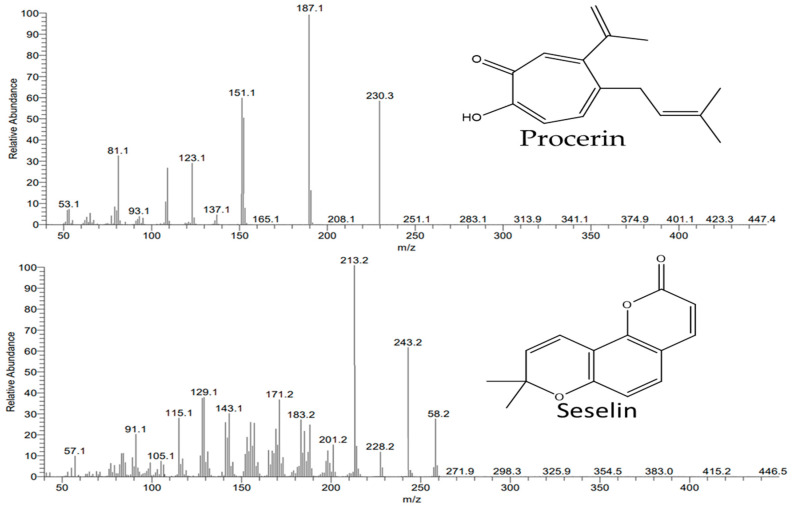
EI-MS spectra of the two compounds, procerin (Rt: 35.68) and seselin (Rt: 40.28).

The predominant constituents of this oil were identified as sesquiterpenes, with a relative concentration of 42.19%. Sesquiterpene hydrocarbons accounted for 33.17% of the whole, while oxygenated sesquiterpenes (9.02%) were identified as being present in low relative concentrations. The total number of identified sesquiterpene hydrocarbons were *trans*-caryophyllene and *cis*-cadina-1,4-diene (cubenene), with corresponding relative concentrations of 23.73% and 9.44%. The documented findings of Kanaani and Mohamadi Sani (2015) [26], who revealed that these compounds are present in the EO obtained from *Sonchus arvensis* but as minor constituents, were fully supported by the current data. The two sesquiterpene hydrocarbons, *trans*-caryophyllene and cubenene, were commonly distributed in the EOs of several plants such as *Guibourtia ehie* (A. Chev.) J Léonard and *Oricia suaveolens* (Engl.) Verd. [27], *Xanthium strumarium* [28], and *Gnaphalium elegans* [29]. The oxygenated sesquiterpenes were represented in this study by only two compounds, procerin and curcumenol, with relative concentrations of 6.95% and 2.07%, respectively. Procerin was a rarely reported compound in the EOs derived from plants, including *Atractylodes macrocephala* Koidz [30] and *Salvia rosmarinus* Spenn. [31]. The EI-MS spectral chromatogram of the rare compound procerin is presented in Figure 3.

From all identified constituents, the hydrocarbons were found to have a high relative concentration of 28.77%. The abundance of the hydrocarbons completely agreed with the published data on the EOs derived from *Sonchus arvensis* [26]. *n*-Nonadecane (28.77%) was the only identified hydrocarbon compound. *n*-Nonadecane represented a widely distributed compound in the EOs derived from plants such as Iranian *Rosa × damascena* Herrm [32,33], *Trigonella elliptica* [34], Tunisian *Allium nigrum* L. [35], Brazilian *Anredera cordifolia* Ten. [36], and Egyptian *Centaurea calcitrapa* L. [37].

Other non-terpenoid compounds were categorised as cyclic ketones (20.17%), cyclic benzenoids (2.61%), and fatty acid esters (1.12%). The *trans*-methyl dihydrojasmonate (19.55%), *trans*-jasmonol (0.60%), and *γ*-nonalactone (0.02%) were the only identified cyclic ketones. *trans*-methyl dihydrojasmonate was described as a significant bioactive compound in several plants, including *Kitaibelia balansae* [38] and *Litsea petiolata* Hook. f. [39]. Five benzenoid compounds were identified within seselin as major compounds (1.72%) in addition to minor relative concentrations of benzyl benzoate, vanillin, benzyl acetate, and *p*-methylanisole. Also, the fatty acid esters were found in *S. oleraceus’s* EO with low relative concentrations of only one compound, isopropyl myristate. Carotenoid-derived compounds and monoterpenes were discovered to be minor components, with relative amounts of 1.83% and 0.74%, respectively. The two isomers of damascene, *Z*-*β*-damascone (1.78%) and *Z*-*α*-damascone (0.03%) were the carotenoid components that were found overall. Conversely, a total of five compounds were identified as monoterpene components, with α-thujaplicin (0.66%) being the predominant component and four additional compounds being detected as minors (0.01–0.03%). Seselin was detected as a main benzenoid in the EO derived from some plants, including *Clausena excavate* Burm.f. [40] and *Carum roxburghianum* [41], and a minor component in the EO of *Clausena harmandiana* (Pierre) Guillaumin [40]. The EI-MS spectral chromatogram of this identified compound, seselin, is presented in Figure 3.

The qualitative and quantitative differences between the EO of *S. oleraceus’s* above-ground parts in the present work and the previous data of other *Sonchus* plants might be directly ascribed to the differences between the plant itself and the plant parts, in addition to the climatic conditions of the collection area for each plant [13].

### 3.2. Antioxidant Activity

**Figure 4:**
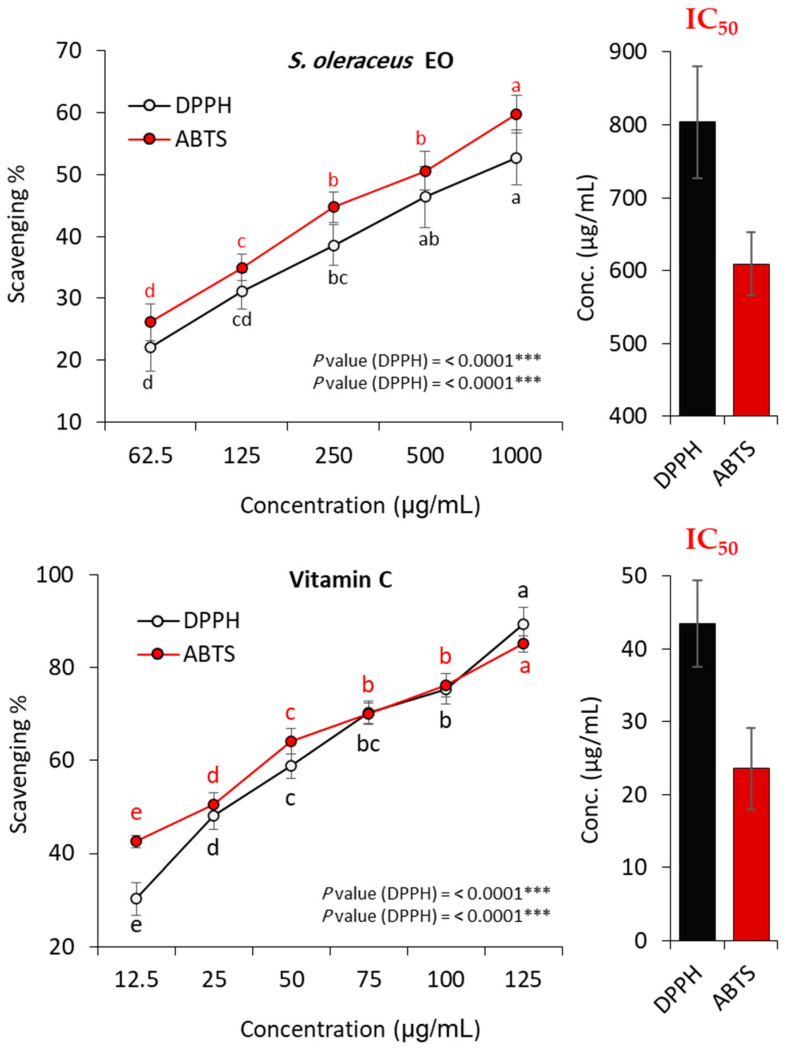
The antioxidant capacity of EO from *S. oleraceus*’s above-ground sections was assessed using DPPH and ABTS tests at several dosages in comparison to the standard (ascorbic acid). *** Different letters inside each line curve indicate values significant at *p* 0.05 following Tukey’s HSD test.

Using DPPH and ABTS assessments, the potential for antioxidant activity of the EO of *S. oleraceus*’s above-ground parts was studied, and its results were contrasted with those of ascorbic acid as the standard antioxidant therapy. The results demonstrated the oil’s dose-dependent capacity to scavenge the DPPH radicals, as shown in Figure 4. With the EO dosages of 62.50, 125.00, 250.00, 500.00, and 1000.00 µg/mL, correspondingly, the DPPH colours were reduced to 22.14%, 44.30%, 31.23%, 38.61%, 46.35%, and 52.71%. Meanwhile, the ascorbic acid concentrations of 12.50, 25.00, 50.00, 75.00, 100.00, and 125.00 µg/mL reduced the colour to 30.29%, 48.01%, 58.76%, 70.31%, 76.37%, and 89.22%, respectively.

According to the ABTS assay results, the EO behaved as a dose-related scavenger in a similar scenario. Using oil concentrations ranging between 62.50, 125.00, 250.00, 500.00, and 1000.00 µg/mL, respectively, the ABTS colours dropped to 26.18%, 34.95%, 44.72%, 50.57%, and 59.74%. The ABTS colours were similarly diminished in the range of 42.60 to 85.03% (Figure 4), with dosages that varied from 12.5 to 125.0 µg/mL for the reference standard.

Consequently, the EO showed DPPH- and ABTS-scavenging functions, with IC_50_ values of 609.35 and 804.16 µg mL^−1^, respectively (Figure 4). Conversely, ascorbic acid displayed IC_50_ values of 43.39 and 23.55 µg mL^−1^, respectively. These findings revealed that this oil has good free-radical-scavenging capacities compared with ascorbic acid, in addition to comparing this with the documented antioxidant results of EOs of other plants [42,43,44,45]. The current results demonstrate the antioxidant abilities of *S. oleraceus’s* EO that were directly attributed to the chemical constituents, especially the main components. Through synergistic and/or single routes, the primary components of plant extracts and EOs served as radical scavengers [46,47]. The scavenging of DPPH and ABTS radicals depends upon the electron accepting [48,49] and transferring [50] routes, respectively.

The free-radical-scavenging action of antioxidants is generally explained by three methods, including hydrogen proton transfer (HAT), sequential proton loss electron transfer (SPLET), and single electron transfer (SET-PT) [51]. Herein, the oxygenated components within a remarkable concentration of this oil, ≈27%, acted as antioxidant agents via the free electrons on the oxygenated groups that increased the trapping of the free radicals. Additionally, the non-oxygenated constituents made a strong contribution to this activity by a singular and/or synergetic effect. *trans*-caryophyllene is a frequently encountered sesquiterpene present in several plant extracts and EOs, possessing associated antioxidant characteristics [52,53]. Dahham and his co-workers found that *trans*-caryophyllene is a very active antioxidant agent with potent scavenging capacities of both BPPH and FRAP radicals [53]. Furthermore, Eos extracted from *Vernonia chalybaea* Mart. ex DC. [54], *Acquilaria crassna* [53], *Murraya paniculata* [55], *Melissa officinalis* subsp. officinalis [56], and *Tabernaemontana catharinensis* A. DC. [57] were instances of potent antioxidant EOs that included *trans*-caryophyllene as a crucial component that scavenges radicals. Additionally, the main components, including *trans*-methyl dihydrojasmonate, *n*-nonadecane, and procerin, represented active radical-scavenging components of several EOs in some plants like *Murraya paniculata* [55], *Melissa officinalis* subsp. officinalis [56], *Tabernaemontana catharinensis* A. DC. [57], and *Kitaibelia balansae* [38].

### 3.3. Cytotoxic Effect

Utilising the conventional MTT process [58], the cytotoxic impact of EO on *S. oleraceus*’s above-ground parts against the cell growth of HepG2 cell lines was assessed. The procedure consisted of experimenting with the EO at increasing concentrations (0.1, 1.0, 10.0, and 100.0 μg/mL) for 48 h and counting the entire number of living cells. The results (Table 2) revealed that the higher concentration of the EO at 100.0 μg/mL exhibited the highest cell viability of the cell lines within a value of 61.09%. Also, the current data demonstrated that this oil shows a dose-dependent activity against the cell growth of the HepG2 cell lines. The two doses at 1.0 and 0.1 μg/mL exhibited very low effects on the cell growth due to the cell viability of the cell lines within respective percentages of 90.45 and 94.03%. Table 2 displays the EO’s concentration sufficient to inhibit 50% of the cell growth (IC_50_). The findings demonstrated that this oil exhibits moderate cytotoxic potency within the value of IC_50_ at 136.02 µg/mL compared with the reference drug, doxorubicin, within IC_50_ at 0.23 ± 0.01.

The moderate cytotoxic effect of this oil on the growth of HepG2 cancer cells might be ascribed to the chemical constituents, especially the major compounds such as *trans*-caryophyllene, *trans*-methyl dihydrojasmonate, *n*-nonadecane, and procerin. The main trait of these compounds was their ability for electron donation and/or electron transfer, which can cause a nucleophilic attack on the DNA of the cancer cells and thus inhibit or limit cell growth [58]. Based upon this fact, the inhibition of cell growth by these components was ascribed to their ability to donate electrons and thus cause the nucleophilic attack. The abilities of these compounds to have free electrons might be attributed to the following: (i) the presence of the double bonds in *trans*-caryophyllene and *cis*-cadina-1,4-diene; (ii) the oxygenation in the *trans*-methyl dihydrojasmonate (-C=O and -COOCH_3_); and (iii) the oxygenation and double bonds of procerin. The EOs of these plants, including some or all of these compounds as major components, were described as active against the growth of several cancer cell lines. The EOs of some plants, like *Salvia officinalis* [59], *Zornia brasiliensis* [60], and *Zanthoxylum nitidum* [61], were reported to be strong inhibitors of the cell growth of numerous cancer cells, including HepG2 cancer cells, due to the high concentrations of some or all of these compounds. Additionally, HepG2 cancer cell lines are susceptible to the cytotoxic effects of some of these compounds, such as *trans*-caryophyllene. *trans*-caryophyllene affects a number of key pathways in the pathophysiology of tumours, including those involving MAPK, PI3K, Akt, mTOR, S6 kinase 1 (S6K1), and the Signal Transducer and Activator of Transcription (STAT3) [62,63].

## 4. Conclusions

*Sonchus oleraceus* is an important edible plant within several traditional medicines in the Mediterranean area. The current findings represent a key to using the *S. oleraceus* EO and its major compounds as antioxidant and cytotoxic agents. The results of bioassays revealed that this oil exerts radical scavenger abilities of the radicals DPPH and ABTS. Also, this oil demonstrated moderate anti-proliferation via the inhibition of the cancer cells’ growth of HepG2. These bioactivities might be attributed to the chemical components of this oil, especially the main compounds, including *n*-nonadecane, *trans*-caryophyllene, *trans*-methyl dihydrojasmonate, and *cis*-cadina-1,4-diene. The mechanism of the actions of this oil and/or its main compounds as radical scavengers and cytotoxic agents should be studied.

## Figures and Tables

**Table 1:** Chemical components of essential oil derived from *Sonchus oleraceus.*

No	Rt	Rel. Conc. ± SD	Component Name	KI_Rep_	KI_Cal_.	Class
1	3.26	0.01 ± 0.00	*p*-Methylanisole	1018	1015	Benz.
2	5.55	0.01 ± 0.00	Eucalyptol	1031	1029	OM
3	5.80	0.02 ± 0.00	*Z*-β-Ocimene	1037	1035	MH
4	8.02	0.02 ± 0.00	Linalool	1096	1093	OM
5	11.37	0.02 ± 0.00	Benzyl acetate	1162	1160	Benz.
6	18.32	0.60 ± 0.01	*trans*-Jasmonol	1324	1327	CYK
7	18.84	0.03 ± 0.01	*Z*-α-Damascone	1358	1355	Car.
8	18.94	0.02 ± 0.00	*γ*-Nonalactone	1361	1364	CYK
9	19.73	0.03 ± 0.00	Geranyl acetate	1381	1380	OM
10	20.5	1.78 ± 0.04	*Z*-β-Damascone	1387	1389	Car. Der.
11	22.83	0.55 ± 0.03	Vanillin	1394	1396	Benz.
12	22.94	0.66 ± 0.01	*α*-Thujaplicin	1411	1410	OM
13	27.56	23.73 ± 0.25	*trans*-Caryophyllene	1419	1419	SH
14	28.21	0.14 ± 0.01	Ethyl vanillin	1454	1457	Benz.
15	28.36	0.02 ± 0.00	methyl-*γ*-Ionone	1481	1483	Car. Der.
16	29.27	9.44 ± 0.19	*cis*-Cadina-1,4-diene (cubenene)	1495	1497	SH
17	29.68	19.55 ± 0.22	*trans*-Methyl dihydrojasmonate	1682	1685	CYK
18	30.68	2.07 ± 0.08	Curcumenol	1734	1731	OS
19	33.98	0.17 ± 0.01	Benzyl benzoate	1760	1758	Benz.
20	34.28	1.12 ± 0.05	Isopropyl myristate	1829	1831	FA-E
21	35.32	28.77 ± 0.32	*n*-Nonadecane	1900	1901	HC
22	35.68	6.95 ± 0.12	Procerin	1931	1934	OS
23	40.28	1.72 ± 0.06	Seselin	1998	2001	Benz.
		2.61	Benzenoids (Benz.)			
		20.17	Cyclic ketones (CYKs)			
		1.12	Fatty acid esters (FA-Es)			
		28.77	Nonterpenoidial hydrocarbons (HCs)			
		0.02	Monoterpene hydrocarbons (MHs)			
		0.72	Oxygenated monoterpenes (OMs)			
		33.17	Sesquiterpene hydrocarbons (SHs)			
		9.02	Oxygenated sesquiterpenes (OSs)			
		1.83	Carotenoid-derived compounds (Car. Der.)			
		97.43	Total identified			

Rt: Retention times, KI_Lit_: reported, and KI_Exp_.: calculated Kovats indexes. Authentication was accomplished by comparing the retention indices and components’ mass spectral data with the NIST Mass Spectral Library, the Wiley Registry of Mass Spectral Data (8th edition, 2), and the literature [25].

**Table 2:** Cytotoxic effect (cell viability % and IC_50_) of EOs of *S. oleraceus*’s above-ground parts on HepG2 cancer cells.

	EO Concentration (μg/mL)			
Cell viability %	100.0	10.0	1.0	0.1
	61.09	80.26	90.45	94.03
IC_50_ (µg/mL)				
*S. oleraceus*’s EO		136.02 ± 1.27		
Doxorubicin (Reference drug)		0.23 ± 0.01		

## Data Availability

Data is contained within the article.
